# Supplementary material for: Chromothripsis during telomere crisis is independent of NHEJ, and consistent with a replicative origin
Source: Genome Res. 2019 May;29(5):737–49. doi: 10.1101/gr.240705.118 (PMC6499312; doi:10.1101/gr.240705.118)
Supplement: Supplemental Material [file supp_gr.240705.118_Supplemental_file_1.zip › contigs/annotated_contigs/DB93/contig.2.DB93_length_480_mean_cov_4.0625.docx]

**DB93_length_480_mean_cov_4.0625**

```
CGGTGGTCTTTCAGTCATGCAGAAAATCCCAATATTATAATATTTATCTATGCCTATTATCAAT|AGATTTGAAACTAGCTATTAAATG
                                                                 >chr3:81764682-81764854
AGTTGTTTCATATATGGAATTACTCTAAGACTAATTGGTATATACCACCTACTATGTGCCAGGCATGGTACTACACAAGAAAGAATAGA
 + E=6e-92
TTCACAAATAAAAGACTGAGTTATATATTGCTAGTCCCATTAGCAACTGACCTTCAATT|CCTC|TTTATTCCAGGATTTGCCTCTGAA
                                                                >chr16:69680409-6968064
GCAGGGAGTGTCTGCATTAAAAATGACCTGTAGTTCTCTGCTTCATAGGTTAGAAACTTATTTTAATATTTTGTGGCTAAGCACAGTCC
9 + E=7e-133
CACTTTTCAAATTCTATAATGAATTATTTAATTGGCATTGAATGTGGACCACCAGCATATATTTCATATTGGGGTTTTCTTTATTACTT

TAAATGATTAAAATTCAATAGAATGTCCAGCTGAGGCA
```
